# Dynamic Metabolic Response to (p)ppGpp Accumulation in *Pseudomonas putida*

**DOI:** 10.3389/fmicb.2022.872749

**Published:** 2022-04-14

**Authors:** Philippe Vogeleer, Fabien Létisse

**Affiliations:** INSA, UPS, Toulouse Biotechnology Institute, Université de Toulouse, Toulouse, France

**Keywords:** metabolome, metabolomics, metabolic regulation, purine pathway, metabolic control, ppGpp

## Abstract

The stringent response is a ubiquitous bacterial reaction triggered by nutrient deprivation and mediated by the intracellular concentrations of ppGpp and pppGpp. These alarmones, jointly referred to as (p)ppGpp, control gene transcription, mRNA translation and protein activity to adjust the metabolism and growth rate to environmental changes. While the ability of (p)ppGpp to mediate cell growth slowdown and metabolism adaptation has been demonstrated in *Escherichia coli*, it’s role in *Pseudomonas putida* remains unclear. The aims of this study were therefore to determine which forms of (p)ppGpp are synthetized in response to severe growth inhibition in *P. putida*, and to decipher the mechanisms of (p)ppGpp-mediated metabolic regulation in this bacterium. We exposed exponentially growing cells of *P. putida* to serine hydroxamate (SHX), a serine analog known to trigger the stringent response, and tracked the dynamics of intra- and extracellular metabolites using untargeted quantitative MS and NMR-based metabolomics, respectively. We found that SHX promotes ppGpp and pppGpp accumulation few minutes after exposure and arrests bacterial growth. Meanwhile, central carbon metabolites increase in concentration while purine pathway intermediates drop sharply. Importantly, in a Δ*relA* mutant and a ppGpp^0^ strain in which (p)ppGpp synthesis genes were deleted, SHX exposure inhibited cell growth but led to an accumulation of purine pathway metabolites instead of a decrease, suggesting that as observed in other bacteria, (p)ppGpp downregulates the purine pathway in *P. putida*. Extracellular accumulations of pyruvate and acetate were observed as a specific metabolic consequence of the stringent response. Overall, our results show that (p)ppGpp rapidly remodels the central carbon metabolism and the *de novo* purine biosynthesis pathway in *P. putida*. These data represent a hypothesis-generating resource for future studies on the stringent response.

## Introduction

The stringent response is a ubiquitous bacterial reaction to forms of stress such as nutrient deprivation, allowing bacteria to rapidly adapt to their environments. Stringent response is mediated by increases in the intracellular concentrations of guanosine tetraphosphate (ppGpp) and guanosine pentaphosphate (pppGpp), referred to together as (p)ppGpp, which act as intracellular signaling molecules and regulate all basic cellular functions including protein activities and DNA replication, transcription and translation (Hauryliuk et al., 2015).

The regulatory roles and targets of (p)ppGpp are therefore the object of intensive research that was recently comprehensively reviewed by Irving et al. (2021). In principle, adaptive changes such as the stringent response can be mediated by (i) “hierarchical” regulation, changes in the abundance of active proteins through gene-expression alterations and/or covalent modifications (acetylation, phosphorylation, methylation, etc.) of existing proteins and/or mRNA molecules and (ii) “metabolic” regulation, changes in the concentration of metabolites e.g., substrates, products and effectors (activators, inhibitors, allosteric regulators) through direct effects on protein activity (ter Kuile and Westerhoff, 2001). Remarkably, (p)ppGpp regulation occurs at both the hierarchical and the metabolic levels, independently or even in concert.

At the hierarchical level, (p)ppGpp regulates a broad range of metabolic processes by binding directly to RNA polymerase (RNAP). This profoundly alters gene transcription profiles, reducing the expression of genes involved in rRNA and macromolecule (e.g., DNA and phospholipid) synthesis, and increasing the expression of genes involved in the synthesis of amino acids and nutrient transporters (Irving et al., 2021). In Firmicutes, (p)ppGpp has recently been shown to tune gene expression locally by directly binding to a specific class of riboswitches (Sherlock et al., 2018). (p)ppGpp also inhibits several enzymes involved at key stages of mRNA translation (Irving et al., 2021), including initiation, elongation and termination (Vinogradova et al., 2020), (Rojas et al., 1984; Kihira et al., 2012; Zhang et al., 2018). As well as reducing the translation rate, (p)ppGpp alters the formation of active 70S ribosomes by inhibiting small GTPases involved in ribosome maturation (Feng et al., 2014; Corrigan et al., 2016). Altogether, these effects lead to drastically reduced protein production.

By binding to active or allosteric sites (p)ppGpp, also inhibits or enhances the catalytic activity of enzymes involved in metabolic pathways. (p)ppGpp reduces the activity of several enzymes involved in purine metabolism in *E. coli* (Wang et al., 2020), *Bacillus subtilis* (Kriel et al., 2012), *Staphylococcus aureus* (Corrigan et al., 2016), *Enterococcus faecalis* (Gaca et al., 2013) and, *Bacillus anthracis* (Yang et al., 2020). Systematic approaches recently developed in *E. col*i and in *B. anthracis* (Zhang et al., 2018; Wang et al., 2019; Yang et al., 2020) have expanded the list of (p)ppGpp binding proteins. Many enzymes involved in *de novo* purine biosynthesis or purine salvage have been shown to bind (p)ppGpp and to have their catalytic activity markedly reduced by ppGpp *in vitro* (Zhang et al., 2018; Wang et al., 2019). Glutamine amidophosphoribosyltransferase (PRTase) (PurF), hypoxanthine PRTase (Hpt), guanine PRTase (Gpt), inosine-guanosine kinase (Gsk), and the pyrimidine/purine nucleotide 5′-monophosphate nucleosidase (PpnN) have thereby been identified as likely *in vivo* targets in *E. coli* (Wang et al., 2019, 2020; Zhang et al., 2019). In Firmicutes, unlike in most proteobacteria species, (p)ppGpp has been shown to regulate guanylate cyclase (Gmk) (Kriel et al., 2012; Gaca et al., 2013; Corrigan et al., 2016). Conversely, PurF is a (p)ppGpp target in *E. coli* but not in Firmicutes (Yang et al., 2020). While (p)ppGpp protein targets are thus not fully conserved between species, (p)ppGpp’s down-regulatory effect on purine biosynthesis is a common feature of diverse bacteria, indicating that this pathway plays a pivotal role in the stringent response.

In *Pseudomonas spp*., the (p)ppGpp-mediated stringent response is crucially involved in biofilm dispersal (Diaz-Salazar et al., 2017), antibiotics resistance (Nguyen et al., 2011; Khakimova et al., 2013; Stewart et al., 2015; Martins et al., 2018; Durand-Reville et al., 2021), persistence (Verstraeten et al., 2015; Wood and Song, 2020) and virulence (Erickson et al., 2004; Vogt et al., 2011; Mansour et al., 2016; Xu et al., 2016; Pletzer et al., 2017). However, the impact of (p)ppGpp accumulation, notably at the metabolic level, has gained less attention in Pseudomonas spp. than it has in *E. coli* or *B. subtilis*. In this study, we investigated the metabolic response of *P. putida* to severe intracellular (p)ppGpp accumulation, quantifying the exometabolome by NMR and a broad spectrum of intracellular metabolites by mass spectrometry. (p)ppGpp accumulation was triggered by treating mid-exponential cultures of *P. putida* with serine hydroxamate (SHX), a serine analog known to have this effect in *E. coli* (Patacq et al., 2020). The regulatory effects of (p)ppGpp were characterized by measuring the concentration variations in intra- and extracellular metabolites induced by (p)ppGpp stimulation, focusing on the short-term response. Interestingly, we found that ppGpp and pppGpp both began to accumulate within a few minutes of SHX induction and following the same dynamic profile. Cell growth was arrested but the cells remained metabolically active. Similar experiments performed on a *P. putida* Δ*relA* mutant and *P. putida* ppGpp^0^ strain which do not produce (p)ppGpp (Sze et al., 2002) confirmed that growth arrest is induced by SHX, not the stringent response, and that (p)ppGpp is essential to overcome the growth disruption caused by SHX. Comparing intra- and extracellular metabolites between the wild-type and mutant strains suggests that the main metabolic effect of (p)ppGpp is a downregulation of purine metabolism, with PurF and PurA as potential *in vivo* binding targets for (p)ppGpp in *P. putida*.

## Materials and Methods

### Bacterial Strain and Culture Conditions

Wild-type (WT) *Pseudomonas putida* KT2440 and the Δ*relA* and ppGpp^0^ derivative strains used in this study were kindly provided by Fernando Govantes (Centro Andaluz de Biología del Desarrollo, Universidad Pablo de Olavide) (Diaz-Salazar et al., 2017) (see Supplementary Table 1). All strains were stored at −80°C in LB medium containing 15% (vol/vol) glycerol. For all experiments, the strains were first streaked onto LB agar plates (with 50 μg/ml kanamycin for the Δ*relA* and ppGpp^0^ mutants) and incubated overnight at 30°C. A 3 mL sample of LB medium (containing 50 μg/ml kanamycin when required) was then inoculated from a single isolated colony and incubated at 30°C and 200 rpm in an orbital shaker (Inova 4230, New Brunswick Scientific, New Brunswick, NJ, United States). After 8 h of incubation, the cells were first diluted (1/1000) in 25 ml of M9 medium containing 3 g⋅L^–1^ glucose, 17.4 g⋅L^–1^ Na_2_HPO_4_ ⋅ 12H_2_O, 3.0 g⋅L^–1^ KH_2_PO_4_, 2.0 g⋅L^–1^ NH_4_Cl, 0.5 g⋅L^–1^ NaCl, 0.5 g⋅L^–1^ MgSO_4_, 3.3 mg⋅L^–1^ CaCl_2_, and 1 ml of a trace element solution containing 15 mg⋅L^–1^ Na_2_EDTA ⋅ 2H_2_O, 4.5 mg⋅L^–1^ ZnSO_4_ ⋅ 7H_2_O, 0.3 mg⋅L^–1^ CoCl_2_ ⋅ 6H_2_O, 1 mg⋅L^–1^ MnCl_2_ ⋅ 4H_2_O, 1 mg ⋅L^–1^ H_3_BO_3_, 0.4 mg⋅L^–1^ Na_2_MoO ⋅ 2H_2_O, 3 mg⋅L^–1^ FeSO_4_ ⋅ 7H_2_O, and 0.3 mg⋅L^–1^ CuSO_4_ ⋅ 5H_2_O. The glucose, MgSO_4_ and trace element solutions were filtered through a 0.2 μm filter (Minisart 0.2 μm syringe filter; Sartorius, Göttingen, Germany) and the other solutions were autoclaved. Since a kanamycin resistance gene was inserted into the genome of the mutants, kanamycin was then not added to the M9 precultures and cultures to avoid any unforeseen effects of the antibiotic. After overnight incubation at 30°C and at 200 rpm, exponentially growing cells were harvested by centrifugation (sigma 3–18K centrifuge, Sigma-Aldrich, Seelze, Germany) at 5,000 g for 5 min at room temperature, washed twice in fresh medium without glucose, and used to inoculate 50 ml of M9 medium with 3 g⋅L^–1^ glucose at an optical density at 600 nm (OD_600_) of 0.15 and incubated at 30°C at 200 rpm. The cells were grown aerobically in baffled shake-flasks in three separate biological replicates and the cultures were monitored at OD_600_ with a Genesys 6 spectrophotmeter (Thermo, Carlsbad, CA, United States). Stringent response was triggered by adding 0.2 mM serine hydroxamate (SHX) to the cell cultures in mid-exponential phase at biomass concentrations of between 0.75 and 1.0 g⋅L^–1^.

### Sampling and Intracellular Metabolite Extraction

Samples and intracellular metabolites were extracted as described previously (Patacq et al., 2020) with some modifications. Briefly, 50 μl of culture was withdrawn 0, 1, 2, 5, 10, 20, 30, 45, 60, 90, 120, 150, and 180 min after SHX induction and added to 1 ml of a precooled methanol-acetonitrile-H_2_O (4:4:2) solution at −20°C to quickly quench metabolic activity (Millard et al., 2014). ^13^C-labeled metabolites cell extract (25 μl, see below) was added as an internal standard (Wu et al., 2005). The samples were then vigorously vortexed, incubated at −20°C for at least 2 h, evaporated overnight to dryness in a SpeedVac (SC110A SpeedVac Plus, ThermoSavant, Waltham, MA, United States) and stored at −80°C until analysis.

### Production of the ^13^C-Labeled Internal Standard Solution

^13^C-labeled metabolites were extracted from *P. putida* KT2440 cells grown in a 500 ml bioreactor (my-Control, Applikon Biotechnology INC, Sunnyvale, CA, United States) containing 200 ml of M9 medium supplemented with 7 g⋅L^–1^ U-^13^C labeled glucose (Euriso-Top, Saint Aubin, France) and containing 1.0 g⋅L^–1^ NH_4_Cl, 0.5 g⋅L^–1^ KH_2_PO_4_, 0.25 g⋅L^–1^ NaCl, 0.25 g⋅L^–1^ MgSO_4_, 3.3 mg⋅L^–1^ CaCl_2_, and 1 ml of the trace element solution described above. The pH was maintained at 7 by automatically adding 14% (g/g) ammonia or 10% (g/g) phosphoric acid and the temperature was set to 30°C. Adequate aeration of the culture was achieved by automatically controlling the stirrer speed and aeration to maintain > 30% oxygen saturation. The stringent response was triggered by adding 0.8 mM of SHX to the culture when the OD_600_ reached 3.5. One hour after SHX exposure, 50 ml of culture was withdrawn from the bioreactor and directly quenched in 200 ml of a precooled (4:4:2) methanol-acetonitrile-H_2_O solution at −20°C. The solution was then vigorously vortexed, incubated at −20°C for 2 h, and evaporated overnight to dryness in a SpeedVac (SC110A SpeedVac Plus, ThermoSavant, Waltham, MA, United States). The ^13^C-metabolites were then resuspended in 50 ml deionized water and stored at −80°C until use.

### Analysis of Extracellular Metabolites by Nuclear Magnetic Resonance

Extracellular metabolites of WT *P. putida* KT2440, and of the Δ*relA* and ppGpp^0^ mutants were identified and quantified by nuclear magnetic resonance (NMR). After SHX induction, 1 ml of broth was collected every 30 min, centrifuged at 14,500 g for 3 min, and the supernatants were stored at −20°C until analysis. The supernatants were mixed with 20 μl of an internal standard consisting of D_2_O and 2.35 g/l deuterated trimethylsilylpropanoic acid (TSP-d4). Proton NMR spectra were recorded on an Avance III 500-MHz spectrometer equipped with a 5-mm z-gradient BBI probe (Brucker, Rheinstatten, Germany). Quantitative ^1^H NMR analysis was performed at 286 K, using a single pulse and a relaxation of 10 s. Thirty-two scans were accumulated (32 k data points with a spectral width of 10 ppm) after four dummy scans. The spectra were processed and metabolites were quantified using Topspin 3.1 (Bruker, Rheinstatten, Germany). Three separate biological replicates were analyzed in this way for each strain.

### Analysis of Intracellular Metabolites by IC-ESI-HRMS

After resuspension of the cell extract samples in 50 μl of water, cell debris were removed by centrifugation at 14,500 g for 10 min at 4°C. The samples were then analyzed using an ion chromatograph (IC; Thermo Scientific Dionex ICS- 50001 system; Dionex, Sunnyvale, CA, United States) coupled to an LTQ Orbitrap mass spectrometer (Thermo Fisher Scientific, Waltham, MA, United States) equipped with a heated electrospray ionization (ESI) probe. The ion chromatography method was adapted from Patacq et al. (2018). The KOH gradient was modified as follows: 0.5 mM for 1 min; 0.5 to 4.1 mM from 9.5 to 14.6 min; 9.65 mM at 24 min; 60 mM at 36 min; 60 to 90 mM from 36.1 to 43 min; 90 to 100 mM from 43.1 to 48 min; and 100 to 0.5 mM from 48.1 to 55 min. The total analysis time was 55 min and the injected volume was 15 μL. Fourier transform mass spectra were recorded in full-scan negative ion mode at a resolution of 60,000 (at m/z 400), with the following source parameters: a capillary temperature of 350°C, a source heater temperature of 350°C, a sheath gas flow rate of 50 AU (arbitrary units), an auxiliary gas flow rate of 5 AU, an S-lens RF level of 60%, and an ion spray voltage of 2.7 kV. Metabolites were identified based on their m/z values with a tolerance of 5 ppm. The data were acquired using the Xcalibur software (Thermo Fisher Scientific, Waltham, MA, United States) and analyzed using TraceFinder 4.0 (Thermo Fisher Scientific, Waltham, MA, United States). Three samples from three independent biological replicates were analyzed.

### Calculation of Growth Rates and Extracellular Fluxes

Growth rates and extracellular metabolite fluxes including glucose, gluconate, ketogluconate, acetate, pyruvate and valine exchange (production and/or consumption) were all calculated from the NMR data. All calculations were performed using Physiofit 1.0.2 (Peiro et al., 2019), a program designed to estimate growth rates and exchanges fluxes from time-course cultures of microorganisms grown under metabolic (pseudo) steady-state. Extracellular metabolite fluxes were determined from the rates of disappearance (or appearance) of substrates and products, in culture supernatants, as measured by NMR.

### Data Analysis

Metabolite concentration changes (expressed as mean log_2_ ratios with respect to the WT strain) were clustered based on the Pearson correlation between metabolites using MEV v4.0.9.

## Results

### Dynamic Response to Serine Hydroxamate Addition

**FIGURE 1:**
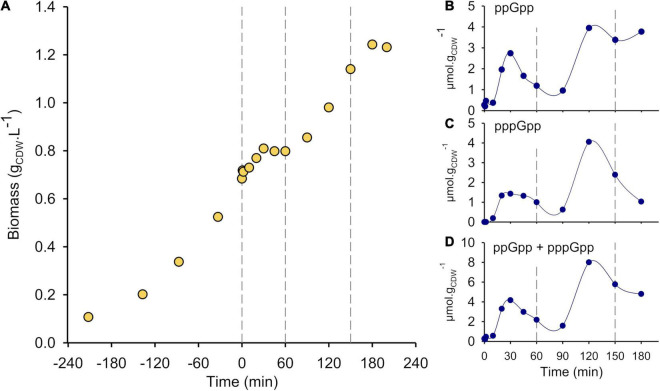
SHX addition leads to severe growth inhibition and triggers ppGpp and pppGpp synthesis. **(A)** Growth curve of wild type *P. putida* KT2440 in M9 medium supplemented with 3 g⋅L^–1^ glucose. SHX (0.2 mM) was added at *t* = 0 min. The growth phases are delimited by vertical dashed lines: before *t* = 0, initial exponential growth; from 0 to 60 min, growth arrest; from 60 to 150 min, second growth phase; after 150 min, stationary phase. Intracellular concentrations (micromoles per gram of cell dry weight—μmol g_CDW_^–1^) of **(B)** ppGpp, **(C)** pppGpp and **(D)** (p)ppGpp (ppGpp + pppGpp) from 0 to 180 min. The results obtained from the other biological replicates are shown in Supplementary Figure 1.

**FIGURE 2:**
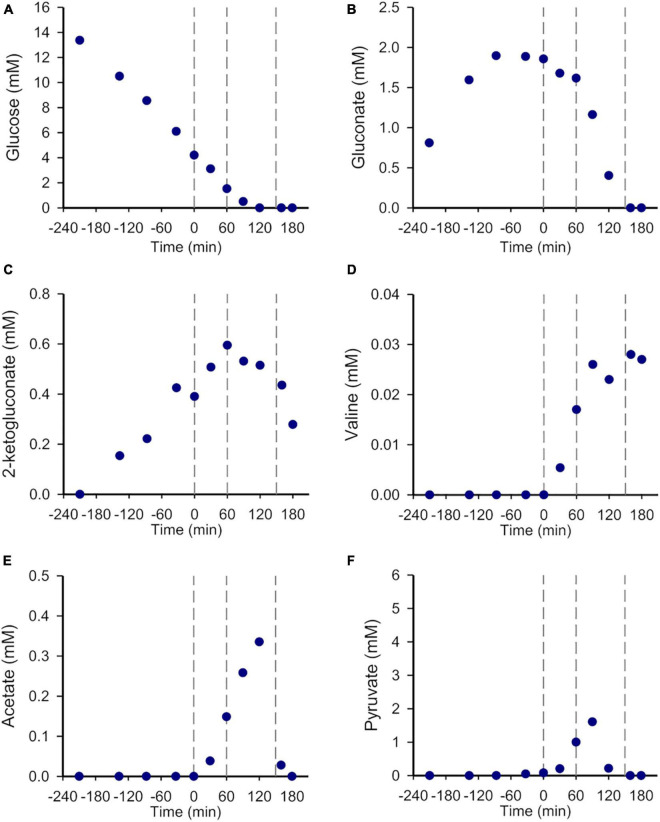
The metabolism of *P. putida* KT2440 is dramatically altered but remains active after SHX addition. Time-evolutions of **(A)** glucose, **(B)** gluconate, **(C)** 2-ketogluconate, **(D)** valine, **(E)** acetate and **(F)** pyruvate concentrations (mM). The vertical dashed lines delimit the different growth phases (see Figure 1). The results obtained from the other biological replicates are shown in Supplementary Figure 2.

**FIGURE 3:**
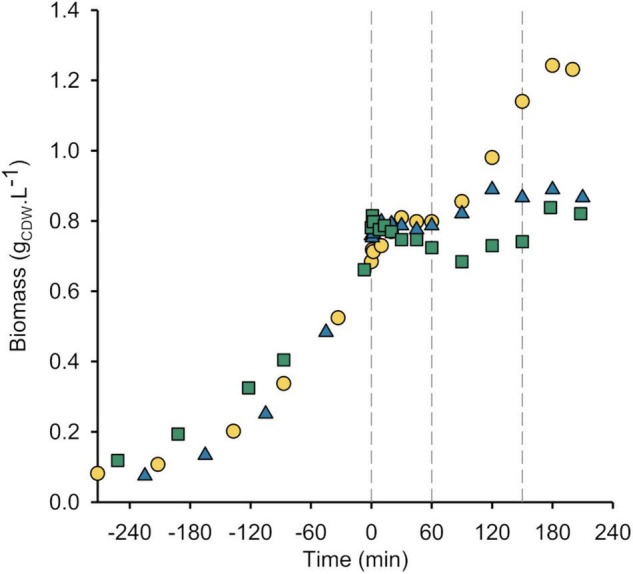
Growth is inhibited by SHX, not (p)ppGpp. Growth curves of wild-type *P. putida* KT2440 (yellow circle), Δ*relA* mutant (blue triangle) and ppGpp^0^ strain (green square). The vertical dashes delimit the different growth phases defined for the WT strain (see Figure 1). The results obtained from the other biological replicates are shown in Supplementary Figure 4.

**FIGURE 4:**
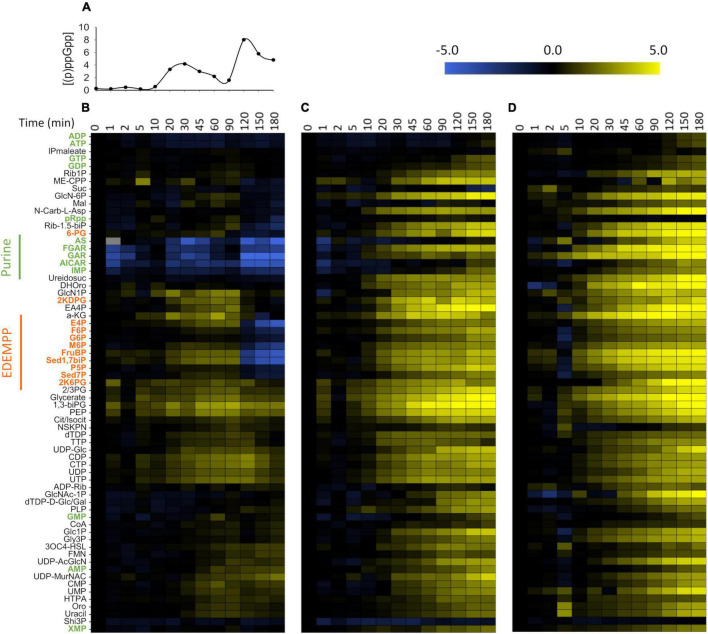
Global metabolic response to SHX addition. Intracellular concentration profile of (p)ppGpp in WT *P. putida* KT2440 **(A)** and variations in metabolite levels in response to SHX addition in **(B)** wild-type (WT) *P. putida* KT2440, **(C)** the Δ*relA* mutant and **(D)** the ppGpp^0^ strain. The columns correspond to different time points just before (*t* = 0 min) and after SHX addition. Metabolome dynamics were determined from LC-MS ^12^C/^13^C quantitative data on 67 metabolites. Fold-changes (log_2_) were calculated relative to exponentially growing cells. Metabolites in the WT strain were hierarchically clustered based on Pearson correlation coefficients. For simplicity, the metabolites are displayed in the same order for the Δ*relA* and ppGpp^0^ strains as for the WT. The data shown were extracted from the same biological replicate as shown in Figures 1, 2. The corresponding results for the two other biological replicates are shown in Supplementary Figure 6 and the log_2_ ratios of the relative metabolite concentration changes for the three biological replicates are given in supplementary material. Non-canonical abbreviations are given in the legend of Supplementary Figure 6.

**FIGURE 5:**
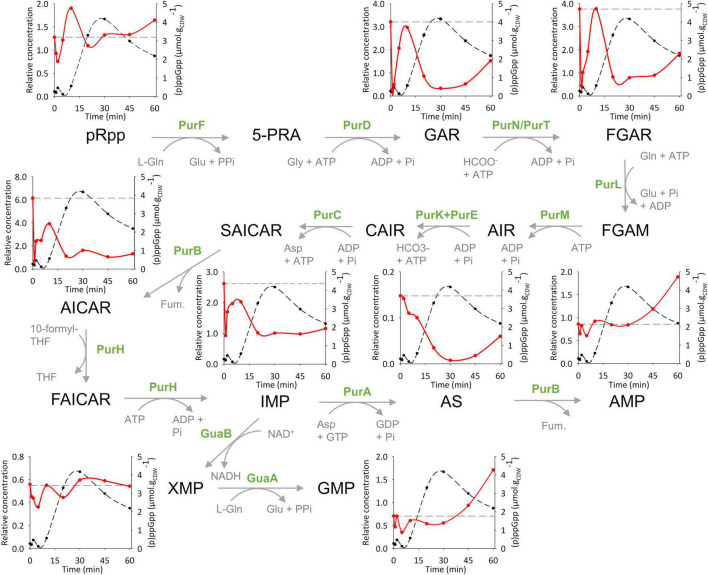
(p)ppGpp acts as a metabolic regulator of the *de novo* purine synthesis pathways in *P. putida*. Metabolic pathway scheme for *de novo* purine synthesis. The inset graphs display the short-term concentration variations of pRpp, GAR, FGAR, AICAR, IMP, AS, AMP, XMP and GMP in wild-type *P. putida* KT2440 (red line) during the first phase of (p)ppGpp accumulation (black dots) in response to SHX addition. Metabolite concentrations are determined relative to the concentration of their U-^13^C labeled forms. The dashed gray lines on each graph show the baseline levels of the metabolites (i.e., intracellular levels before SHX addition). The results obtained from the other biological replicates are shown in Supplementary Figure 7. Non-canonical abbreviations: pRpp: phosphoribosylamine; 5-PRA: 5-phosphoribosylamine; GAR: 5′-phosphoribosylglycinamide; FGAR: 5′-Phosphoribosyl-*N*-formylglycinamide; FGAM: 5′-Phosphoribosyl-*N*-formylglycinamidine; AIR: 5′-Phosphoribosyl-5-aminoimidazole; CAIR: 5′-Phosphoribosyl-4-carboxy-5-aminoimidazole; SAICAR: 5′-Phosphoribosyl-4-(N-succinocarboxamide)-5-aminoimidazole; AICAR: 5′-Phosphoribosyl-5-amino-4-imidazolecarboxamide; FAICAR: 5′-Phosphoribosyl-5-amino-4-imidazolecarboxamide; AS: Adenylosuccinate; Fum: Fumarate; Gln: Glutamine; Glu: Glutamate; Gly: Glycine.

The macroscopic effects of SHX addition were characterized in *Pseudomonas putida* KT2440 cells growing exponentially under aerobic conditions in minimal medium with 3 g⋅L^–1^ glucose (16.6 mM) as sole carbon source. Results are reported as the mean ± standard deviation of three separate biological replicates. For clarity, the data of one representative biological experiment out of three are shown in Figures 1–5 and those obtained from the other biological replicates are given in supplementary material.

Before SHX induction, the growth was exponential (μ = 0.57 ± 0.03 h^–1^) and accompanied by gluconate and 2-ketogluconate accumulation as previously reported (Nikel et al., 2015; Kohlstedt and Wittmann, 2019). As expected, the addition of 0.2 mM SHX in mid-exponential phase strongly inhibited growth, but not immediately, as the biomass concentration kept increasing before reaching a plateau at *t* = 60 min (Figure 1A and Supplementary Figure 1A). During that first hour, glucose continued to be consumed at half of the estimated rate during the exponential growth phase (3.97 ± 0.93 mmol g^–1^ h^–1^ *versus* 8.32 ± 1.01 mmol g^–1^ h^–1^, respectively) (Figure 2A and Supplementary Figure 2A), gluconate and 2-ketogluconate were either consumed or excreted (Figures 2B,C and Supplementary Figures 2B,C), and the concentrations of acetate, pyruvate and valine increased sharply (at 0.16 ± 0.04 mmol g^–1^ h^–1^, 0.89 ± 0.19 mmol g^–1^ h^–1^ and 0.06 ± 0.09 mmol g^–1^ h^–1^, respectively). The latter were not detected during the exponential phase (Figures 2D–F and Supplementary Figures 2D–F) or when no SHX was added (Supplementary Figure 3). These results reveal that the cells remained metabolically active after SHX addition, despite severe growth inhibition.

Growth then resumed until *t* ≈ 150 min, at which stage glucose and the other main carbon sources—including gluconate, acetate and pyruvate but not valine or 2-ketogluconate—were fully depleted in the medium. The final biomass concentration was 1.35 ± 0.14 g L^–1^ (Figure 2 and Supplementary Figure 2). Note that the final biomass concentration for the WT strain grown in similar conditions using 3 g L^–1^ glucose was 1.73 ± 0.02 g L^–1^ when no SHX was added (Supplementary Figure 3). The difference in biomass yields (0.45 ± 0.05 g_CDW_ g_Glucose_^–1^ *versus* 0.58 ± 0.01 g_CDW_ g_Glucose_^–1^) can be explained by non-growth-associated glucose consumption consequent to SHX exposure. During the second growth phase, the growth rate was roughly half the value measured in the first exponential growth phase, 0.29 ± 0.05 h^–1^), glucose, gluconate and to a lesser extent 2-ketogluconate are co-consumed, and pyruvate and acetate continued to accumulate before peaking at *t* = 90 min and *t* = 120 min, respectively. In contrast, the concentration of valine increased gradually right until the end of the experiments (Figure 2 and Supplementary Figure 2).

### Intracellular (p)ppGpp Concentration Dynamics

The intracellular concentrations of ppGpp and pppGpp, measured by LC-MS (Patacq et al., 2018; Figures 1B,C), varied strongly throughout the experiments. Starting from 0.28 ± 0.02 μmol⋅g_CDW_^–1^ just before SHX addition, ppGpp began to accumulate about 10 min after SHX addition, with a first peak (2.28 ± 0.42 μmol⋅g_CDW_^–1^) roughly 30 min after SHX addition. The ppGpp concentration then decreased continuously for 60 min (1.05 ± 0.38 μmol⋅g_CDW_^–1^), before increasing once again up to 3.29 ± 1.06 μmol⋅g_CDW_^–1^ at *t* = 150 min. Interestingly, the pppGpp concentration followed the same trend, albeit with a more moderate initial increase, peaking at 1.78 ± 0.34 μmol g_CDW_^–1^ after 30 min and then at 4.25 ± 1.1 μmol g_CDW_^–1^ after 120 min (Figure 1C). The two major phases of alarmone accumulation appear even more clearly when the ppGpp and pppGpp concentrations are pooled (Figure 1D).

In *E. coli*, SHX is well-known to promote (p)ppGpp accumulation and provoke growth arrest (Tosa and Pizer, 1971; Ross et al., 2016) and we showed in a previous study (Patacq et al., 2020) that growth is inhibited by SHX alone, while the stringent response induced by (p)ppGpp accumulation is crucial to overcome the growth disruption caused by SHX. To confirm that this is also the case in *P. putida*, the experiments were repeated with a Δ*relA* mutant and a ppGpp^0^ strain missing the (p)ppGpp synthetase-encoding genes *relA* and *SpoT* and therefore unable to synthesize (p)ppGpp. The latter strain was used to validate results obtained with the Δ*relA* mutant and exclude any effect related to SpoT.

### Serine Hydroxamate Inhibits Growth

The Δ*relA* mutant grew slightly faster than the WT strain (0.61 ± 0.02 h^–1^ *versus* 0.58 ± 0.02 h^–1^) (Supplementary Figures 4A–C). As observed for the WT strain, SHX interrupted the growth of the Δ*relA* mutant (Figure 3). (p)ppGpp was not detected by LC-MS either before or after SHX addition, confirming that SHX inhibited growth by itself, without (p)ppGpp. For the Δ*relA* mutant furthermore, contrary to the WT strain, no second growth phase was observed (Figure 3 and Supplementary Figure 4C); the growth rate estimated from the slight increase in biomass concentration only reached a tenth of the value estimated in the initial exponential phase. This means that the ability of *P. putida* to overcome the growth disruption caused by SHX addition almost completely disappears without the stringent response. However, glucose continued to be consumed at a similar rate as observed during growth arrest for the WT strain (q_glc_ = 4.24 ± 0.73 mmol g^–1^ h^–1^) showing that the cells were metabolically active. They also consumed gluconate and produced 2-ketogluconate. While acetate and pyruvate did not accumulate after SHX addition as observed for the WT strain, valine excretion was much more pronounced in the Δ*relA* mutant than in the WT strain (Supplementary Figure 5, left panel). Together, these results show that while the Δ*relA* cells stopped growing after SHX addition, they remained metabolically active.

As reported previously (Sze et al., 2002), unlike the equivalent *E. coli* strain, the ppGpp^0^ strain of *P. putida* was able to grow in minimal medium without adding any amino acids. The ppGpp^0^ strain’s estimated growth rate was 0.45 ± 0.03 h^–1^ (about 70% of the WT’s estimated growth rate) (Supplementary Figures 4A,D). The ppGpp^0^ strain accumulated gluconate but not 2-ketogluconate during the exponential phase either because of its reduced growth rate or because of its impaired stringent response. As in the Δ*relA* mutant, adding SHX to ppGpp^0^ cultures led to growth arrest and no transient (p)ppGpp accumulation as expected (Figure 3 and Supplementary Figure 4D). Growth did not resume but the cells continued to consume glucose at 2.86 ± 0.85 mmol g^–1^ h^–1^, indicating that they remained metabolically active. After SHX addition, the gluconate concentration plateaued and the concentration of valine increased in the medium (Supplementary Figure 5, right panel). These results confirm that growth inhibition and the metabolic activity of *P. putida* are independent of (p)ppGpp synthesis, whereas the stringent response is crucial for growth resumption.

In summary, these comparative dynamics analyses show that: (i) the first peak in (p)ppGpp accumulation in the WT strain is mediated by RelA-dependent (p)ppGpp synthesis in response to SHX addition, (ii) the cells’ ability to resume growth is conditioned on the stringent response, and (iii) the second peak in (p)ppGpp accumulation, concurrent with carbon source exhaustion, is very likely due to the cells entering the stationary phase (Cavanagh et al., 2010).

### Whole Metabolome View

Having established the macroscopic effects of SHX addition, we investigated the dynamic metabolic response by quantifying metabolites by LC-MS. Quantitative data over the entire course of the experiments were obtained for 67 intracellular metabolites, representing a substantial fraction of *P. putida*’s central carbon metabolism and nucleotides (the other metabolites were below the quantification limit or too unstable to yield reliable information). Metabolite concentrations were determined relative to their U-^13^C labeled forms by isotope dilution mass spectrometry (IDMS) (Mashego et al., 2004). A higher sampling frequency was used in the first hour of the experiments to catch the expected metabolic regulatory effects of (p)ppGpp. The data, expressed as log_2_ ratios of the relative metabolite concentration changes, are given for the three biological replicates in Supplementary Material.

We used hierarchical clustering [Pearson correlation; (Eisen et al., 1998)] to visualize relative changes over time and compare metabolite profiles between strains (Figure 4). Similar clusters were obtained from the independent analyses of biological replicates (see Supplementary Figure 6). Several general conclusions can be drawn from these data. First, in the WT strain, the variations in metabolite concentrations follow the (p)ppGpp accumulation profiles remarkably closely. The metabolite concentrations varied little in the first 5 min of the experiments, when (p)ppGpp concentrations were low. During the first peak in (p)ppGpp accumulation, metabolites in the purine pathway, which formed a remarkably consistent cluster, decreased drastically in concentration while the concentration of certain nucleotides and of metabolites belonging to the upper parts of the Embden-Meyerhof-Parnas (EMP), Entner-Doudoroff pathway (ED) and pentose phosphate (PP) pathways (termed EDEMPP) increased. When (p)ppGpp levels decreased (after *t* = 90 min), purine intermediates returned to the levels observed before SHX addition, whereas almost all other metabolites accumulated in the cells. During the second peak in (p)ppGpp accumulation, the concentrations of purine intermediates dropped further but so did the concentrations of the EDEMPP pathway metabolites, contrary to what was observed during the first (p)ppGpp peak. Since the upper parts of the EMP and PP pathways are fed by phosphorylated glucose (G6P), this is probably a consequence of glucose depletion in the culture medium. Second, the metabolite profiles of the Δ*relA* and ppGpp^0^ strains differ substantially from those of the WT strain. Almost all metabolites increased in concentration after SHX addition, notably the purine intermediates, whose levels decreased at this stage in the WT strain. Third, the variations in metabolite concentrations are less pronounced in the WT than in the Δ*relA* and ppGpp^0^ strains, with, respectively, 28.4, 67.2, and 80.6% of metabolites increasing or decreasing by a factor of 4 or more. This indicates that the stringent response moderates fluctuations in intracellular metabolites levels. In the same vein, the proportion of metabolites whose levels varied by less than a factor of 2 before glucose exhaustion, was much higher in the WT strain (34.3%) than in the Δ*relA* mutant (13.4%) or the ppGpp^0^ strain (4.5%). This set of homeostatic compounds included nucleotides such as XMP, AMP, ADP, ATP, GTP and GDP. A significant part of the metabolome thus remains homeostatic despite the sudden and severe growth disruption.

In summary, the intracellular levels of several metabolites involved in purine synthesis decrease in the first minutes after SHX addition in WT *P. putida*, but not in Δ*relA* or ppGpp^0^ strains. The fact that these changes occur extremely soon after SHX addition suggests that (p)ppGpp exerts a regulatory effect on purine nucleotide biosynthesis. These results are in line with previous studies in *E. coli* (Hochstadt-Ozer and Cashel, 1972; Wang et al., 2019, 2020) and *B. subtilis* (Kriel et al., 2012) and highlight the conserved nature of this form of metabolic regulation in bacteria.

Variations in intracellular metabolite concentrations can be analyzed to identify enzymes whose activity is regulated by (p)ppGpp *in vivo*. The approach consists in examining the response of upstream and downstream metabolic intermediates to a modulation of the enzyme’s activity, provided its abundance does not change dramatically. While SHX addition can affect transcript levels (Durfee et al., 2008), ppGpp has been shown to have a negative effect on ribosome assembly, and thus limits mRNA translation (Li et al., 2018). Provided (p)ppGpp concentrations remain high therefore, enzyme levels should not vary dramatically on short timescales. Accordingly, we interpreted fluctuations in metabolite levels during the first phase of (p)ppGpp accumulation (0 to 60 min) as reflecting the metabolic regulation of the target enzymes by (p)ppGpp, while the later changes were assumed to reflect a more complex interaction of both metabolic and hierarchical regulation.

### Metabolic Control of Purine Biosynthesis by (p)ppGpp

We investigated the regulatory effect of (p)ppGpp on purine biosynthesis in more detail by examining the concentration profiles of the precursors and intermediates of this pathway alongside those of (p)ppGpp during the first hour (Figure 5).

Some of the metabolites in the purine pathway (pRpp, GAR, FGAR, AICAR and IMP) varied sharply in concentration in the first minutes after SHX addition. Strikingly, the corresponding increase in (p)ppGpp concentration was weaker than observed later on (a ∼2-fold increase *versus* a ∼20-fold increase relative to the basal level). At the end of this period (*t* ≈ 10 min), the intracellular levels of most metabolites tended toward the basal value measured before SHX addition. Then, as (p)ppGpp concentration increased in the WT strain, the levels of GAR, FGAR, AICAR and AS, and to a lesser extent that of IMP, decreased sharply, down to 11, 21, 27, 7, and 50% of the levels measured before (p)ppGpp accumulation and the minima coincided with the (p)ppGpp concentration maximum (*t* = 30 min). In the Δ*relA* and ppGpp^0^ strains in contrast, the concentrations of these metabolites are constant (AICAR, AS and IMP) or even increased dramatically (GAR and FGAR) (Supplementary Figure 7). In the meantime, the levels of pRpp, AMP, XMP and GMP varied little in the WT despite strong (p)ppGpp accumulation.

The dramatic decrease in GAR, FGAR and AICAR combined with the invariance of the pRpp concentration can be explained by a reduction in PurF (pRpp synthase) activity, the decrease in downstream metabolites likely reflecting the inhibition of PurF by (p)ppGpp. This result is in line with previous studies in other bacteria where (p)ppGpp has been shown to interact and inhibit PurF *in vitro* (Ahmad et al., 2019; Wang et al., 2019). Our data match remarkably well the changes reported in these studies in pRpp, GAR, FGAR and AICAR levels upon inhibition of purine biosynthesis. Similarly, AS profile is also consistent with the one recently associated with *in vitro* (p)ppGpp inhibition of PurA (AS synthetase) in Firmicutes (Yang et al., 2020) and much earlier in *E. coli* (Gallant and Cashel, 1967; Stayton and Fromm, 1979). This effect may explain the strong decrease in AS upon (p)ppGpp accumulation that was not observed in the Δ*relA* mutant (Supplementary Figure 7).

This analysis identified two enzymes in the purine pathway (PurF and PurA) as potentially controlled *in vivo* by (p)ppGpp in *P. putida*. PurF catalyzes the first step of the *de novo* purine biosynthesis pathway and PurA catalyzes the committed step of AMP biosynthesis (and of *de novo* ATP synthesis) from IMP. (p)ppGpp may therefore exert dual control on purine biosynthesis, acting simultaneously on the committed step and on a metabolic branch of the pathway.

## Discussion

(p)ppGpp is a global regulator of bacterial physiology that acts at the hierarchical level, by binding to RNA polymerase for example, and at the metabolic level, by activating or inhibiting enzymes. Attempts have recently been made to systematically identify interaction targets for (p)ppGpp or related nucleotides (pGpp) (Zhang et al., 2018; Wang et al., 2019; Yang et al., 2020). Interestingly, several (p)(p)pGpp targets are enzymes involved in *de novo* purine biosynthesis or in the purine salvage pathway, supporting previous biochemical studies (Stayton and Fromm, 1979; Pao and Dyes, 1981). Some have been shown to be critical targets of ppGpp *in vivo* (Wang et al., 2019, 2020; Zhang et al., 2019; Yang et al., 2020). While downregulating the purine synthesis and salvage pathways seems to be a conserved role of (p)ppGpp, the targets within these pathways differ between bacterial species (Irving et al., 2021). In *Pseudomomas spp*., the physiological effects of (p)ppGpp accumulation are unclear, particularly at the metabolic level. The aims of this study were therefore to characterize the dynamic response of *P. putida* to severe growth disruption, caused by RelA-mediated (p)ppGpp accumulation (SHX addition) and investigate the impact of this accumulation on bacterial metabolism. We did this by monitoring cell growth and the variations in intra and extra-cellular metabolite concentrations in response to SHX addition by NMR and by mass spectrometry.

Our results demonstrate, first, that the KT2440 WT strain of *P. putida* can overcome SHX-induced growth disruption, resuming growth about 1 h after the perturbation. We had previously observed this recovery in *E. coli* (Patacq et al., 2020). The fact that the Δ*relA* and ppGpp^0^ strains of *P. putida* showed no significant growth after SHX addition indicates that growth resumption is conditioned on the stringent response. As observed previously in *E. coli* (Patacq et al., 2020), SHX triggered the synthesis of both ppGpp and pppGpp. The concentration of ppGpp measured here during exponential growth (0.276 ± 0.012 μmol/g_CDW_) is similar to the value measured under similar conditions by Ankenbauer et al. (2020) (0.1 μmol/g_CDW_). These authors reported a maximum ppGpp concentration of 0.45 μmol/g_CDW_ upon glucose limitation, much lower than the value measured in our study after SHX addition (2.279 ± 0.423 μmol/g_CDW_), suggesting that chemically triggering the stringent response with SHX enhances ppGpp synthesis. The use of SHX also allowed us to accurately quantify pppGpp, whose intracellular concentration appears to be of the same magnitude as ppGpp’s. Importantly, (p)ppGpp did not accumulate in the Δ*relA* mutant after SHX addition, indicating that SpoT synthetase is inactive in this situation and that no other RSH is involved in this response in *P. putida*. This finding is in keeping with previous results in a *P. aeruginosa* PAO1 Δ*relA* mutant, where ^32^P-radiolabeled ppGpp and pppGpp were likewise not detected after SHX induction (Erickson et al., 2004).

Our results also show that it is SHX alone that provokes growth arrest and that during growth arrest, the cells (of all strains, WT, Δ*relA* and ppGpp^0^) remain metabolically active, continuing to consume glucose and excrete acetate, pyruvate and valine. Interestingly, non-growing but metabolically active cells have also been observed in bacterial cultures treated with translational inhibitors (Lempp et al., 2020). For example, metabolic activity has been observed in *E. coli*, *Vibrio cholerae* and *S. aureus* cells exposed to chloramphenicol, tetracycline and linezolid (Lobritz et al., 2015; Díaz-Pascual et al., 2019). One possible explanation is an imperfect coordination of metabolism and protein synthesis (Lempp et al., 2020). Recently, (p)ppGpp has been shown to be crucially involved in coordinating metabolic activities during starvation, to promote survival (Wang et al., 2020). Our results show likewise that after protein synthesis was strongly altered by SHX exposure, the cells remained metabolically active but did not begin to grow again in the absence of (p)ppGpp, underlying its pivotal role in coordinating metabolism and protein synthesis.

The excretion of acetate and pyruvate by *P. putida* is surprising since *Pseudomonas spp.* prioritize the consumption of organic acids including acetate over glucose catabolism (through a carbon catabolite repression strategy termed “reverse catabolite repression control”, in reference to the CCR strategy in *E. coli*) (Yung et al., 2019), pyruvate and glucose being utilized simultaneously (Collier et al., 1996). This may explain why acetate and pyruvate are not commonly observed in the culture media of *Pseudomonas spp.* even if they are produced and excreted. It can therefore be assumed that the accumulation of acetate and pyruvate observed here upon glucose metabolism is due to inhibited utilization of acetate and pyruvate, which are continuously produced and excreted by the cell. Moreover, this inhibition seems to be controlled by (p)ppGpp, because only traces of pyruvate and no acetate were detected in the Δ*relA* mutant and the ppGpp^0^ strain. Further investigations are clearly required to elucidate the regulatory mechanism underlying acetate and pyruvate accumulation. To our knowledge, valine overflow has never previously been reported in *P. putida* or *Pseudomonas spp.* in general, but valine has been detected in the culture media of stationary phase *E. coli* (Valle et al., 2008; Ito et al., 2013) (unpublished data from our laboratory), and at higher levels in biofilms without the stringent response being involved (Valle et al., 2008). We detected valine after SHX addition in *E. coli* culture media unrelated to (p)ppGpp accumulation (Patacq et al., 2020). This phenomenon is therefore not specific to *P. putida* and is probably a consequence of reduced growth, whether or not nutrients are limiting.

We measured the concentration dynamics of the *P. putida* coli metabolome after SHX addition by LC-MS to understand the regulatory effect of (p)ppGpp at the metabolic level. The concentrations of ppGpp and pppGpp increased in two distinct waves, one 30 min after SHX addition and the second 2 h after SHX addition. Hierarchical clustering showed that the main metabolic response mirrored these fluctuations in (p)ppGpp concentration. The observed metabolites that most strongly followed the variations in (p)ppGpp concentration were those involved in the *de novo* purine biosynthesis pathway (GAR, FGAR, AICAR, AS and to a lesser extent, IMP). These metabolites were at their lowest levels when (p)ppGpp peaked. The third minimum observed in the first few minutes after SHX addition corresponded to a more modest but significant threefold increase in the concentration of ppGpp. Increases in intracellular (p)ppGpp concentration may initially be moderated by (p)ppGpp’s ability to bind to a broad range of proteins, present in high abundance. From a general point of view, the fact that the intracellular levels of GAR, FGAR, AICAR, AS and IMP are sensitive to changes in (p)ppGpp concentration is consistent with the known regulation of *de novo* purine biosynthesis by (p)ppGpp in other bacterial species (Irving et al., 2021).

We attempted to identify potential *in vivo* targets of (p)ppGpp in the purine biosynthesis pathway by focusing our analysis on the short-term response to SHX addition, during which hierarchical regulation of the metabolism can be ignored. The increase in (p)ppGpp concentration immediately after SHX addition was large enough to unequivocally link the response profiles of purine pathway intermediates to (p)ppGpp. Thus, (p)ppGpp accumulation led to strong decreases in GAR (∼10-fold), FGAR (∼5-fold), AICAR (∼4-fold) and AS (∼15-fold), consistent with (p)ppGpp inhibition of PurF and PurA. This fits with the recent identification of PurF and PurA as (p)ppGpp binding targets (Wang et al., 2019; Yang et al., 2020) and biochemical studies showing their competitive inhibition by ppGpp *in vitro* (Gallant and Cashel, 1967; Stayton and Fromm, 1979; Ahmad et al., 2019; Wang et al., 2019). The metabolite profiles measured here match remarkably well with the variations in pRpp, GAR, FGAR, AICAR and IMP measured in *E. coli in vivo*, under (p)ppGpp-mediated inhibition of *de novo* purine biosynthesis (Wang et al., 2019). The inhibition of PurF and PurA suggests that (p)ppGpp regulates *de novo* purine biosynthesis in a concerted way. pRpp is at a crossroad of multiple metabolic pathways, including the synthesis of both purine and pyrimidine nucleotides, the amino acids histidine and tryptophan and the redox cofactors NAD(P)^+^. This dual control may explain how homeostasis of this critical metabolite together with monophosphorylated purine nucleotides is maintained in the cells despite the sudden growth arrest and would complement (p)ppGpp’s regulation of purine salvage pathways, which has been demonstrated in other bacteria (Irving et al., 2021).

In summary, this work provides further evidence that (p)ppGpp regulates purine nucleotide biosynthesis. The fact that this regulation appears to be conserved across bacterial species reinforces the idea that purine biosynthesis plays a pivotal physiological role in the stringent response. As well as providing insights into (p)ppGpp’s role in the regulation of metabolism, this work highlights the potential of quantitative metabolomics to identify *in vivo* targets of regulators at the metabolic level. When combined as done here with an analysis of the concentration dynamics of intracellular metabolites in response to a stimulus, this approach can provide a quantitative understanding of cellular metabolic regulation.

## Data Availability Statement

The original contributions presented in the study are included in the article/Supplementary Material, further inquiries can be directed to the corresponding author.

## Author Contributions

PV and FL conceived and designed the study, contributed to the data analysis and interpretation, manuscript revision, read, and approved the submitted version. PV performed the experiments and drew the figures. FL wrote the first draft of the manuscript. Both authors contributed to the article and approved the submitted version

## Conflict of Interest

The authors declare that the research was conducted in the absence of any commercial or financial relationships that could be construed as a potential conflict of interest.

## Publisher’s Note

All claims expressed in this article are solely those of the authors and do not necessarily represent those of their affiliated organizations, or those of the publisher, the editors and the reviewers. Any product that may be evaluated in this article, or claim that may be made by its manufacturer, is not guaranteed or endorsed by the publisher.
