# Supplementary material for: Dynamic Metabolic Response to (p)ppGpp Accumulation in Pseudomonas putida
Source: Front Microbiol. 2022 Apr 14;13:872749. doi: 10.3389/fmicb.2022.872749 (PMC9048047; doi:10.3389/fmicb.2022.872749)
Supplement: Supplementary file 1 [file Data_Sheet_1.PDF]

Supplementary Material

Supplementary Figures and Tables

Supplementary Figures

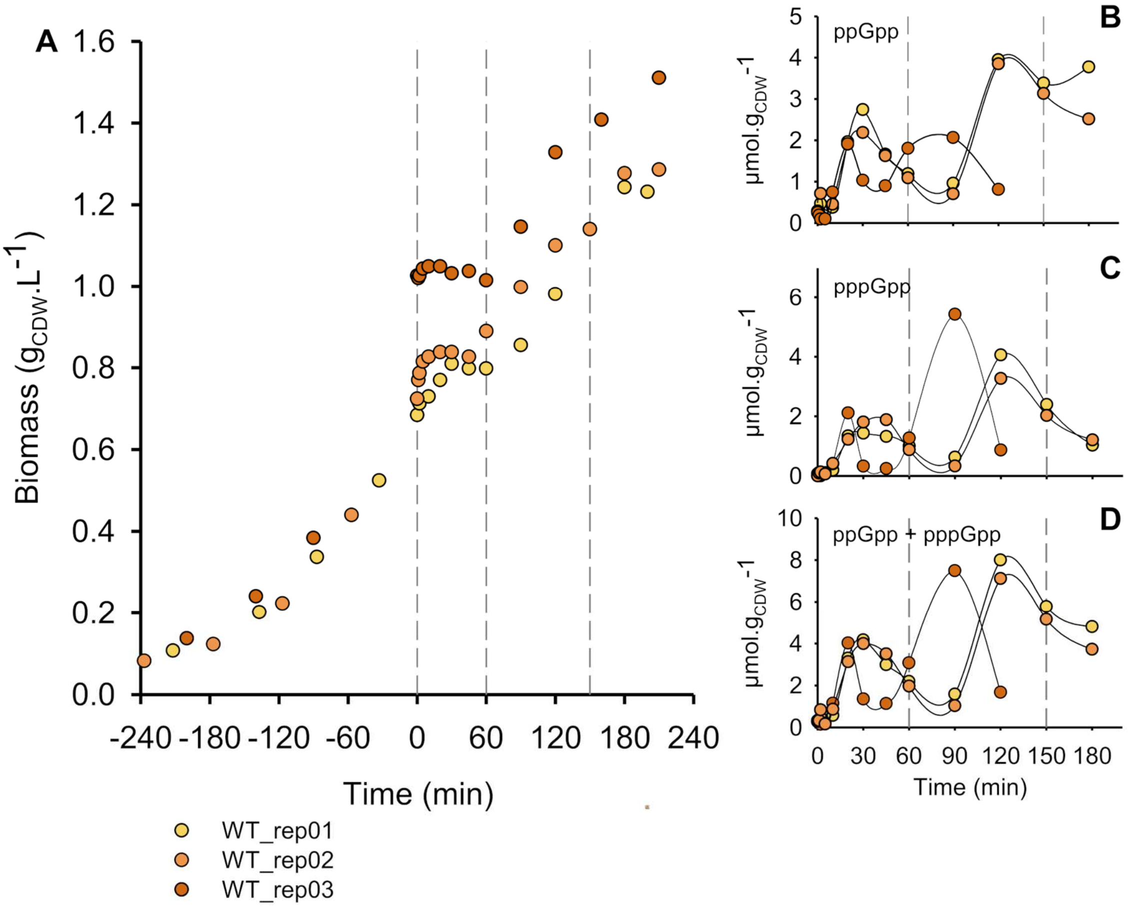

Supplementary Figure S1: Response of wild type *P. putida* KT2440 to SHX addition in the three independent biological replicates. (A) The data from Fig.1 are shown in yellow (rep01), rep02 in orange, and rep03 in dark orange. **(A)** Growth curve of wild type *P. putida* KT2440 cultivated in M9 medium supplemented with 3 g·L^{-1} glucose. SHX (0.2 mM) was added at t= 0 min. The growth phases are delimited by vertical dashed lines: before t = 0, initial exponential growth; from 0 to 60 min, growth arrest; from 60 to 150 min, second growth phase; after 150 min, stationary phase. Intracellular concentrations (micromoles per gram of cell dry weight - µmol.g_{CDW}^{-1}) of **(B)** ppGpp, **(C)** pppGpp and **(D)** (p)ppGpp (ppGpp + pppGpp) from 0 to 180 min.

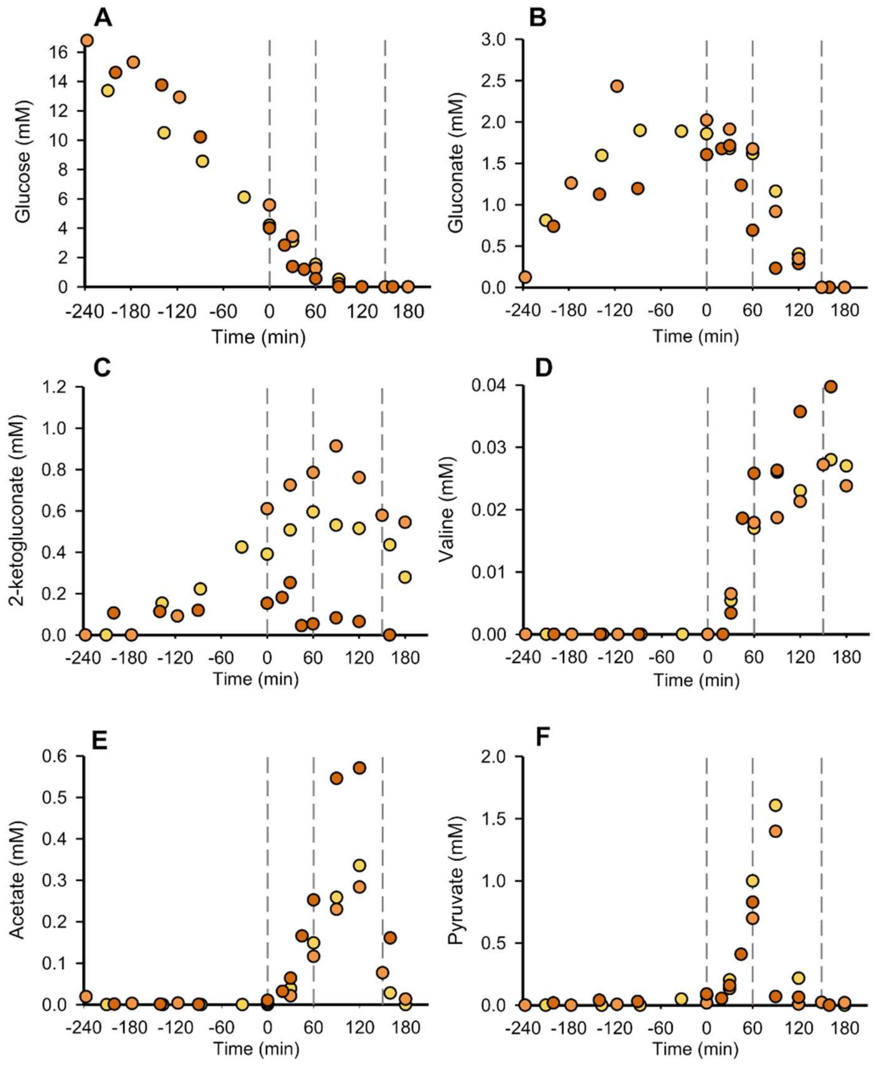

Supplementary Figure S2: Profiles of the extracellular metabolites quantified throughout the cultivations of the wild type *P. putida* KT2440 in the three independent biological replicates. The data from Fig.2 are shown in yellow (rep01), rep02 in orange, and rep03 in dark orange. Time-evolutions of glucose **(A)**, gluconate **(B)**, 2-ketogluconate **(C)**, valine **(D)**, acetate **(E)** and pyruvate **(F)** concentrations (mM) for biological replicate 1 (yellow), 2 (green) and 3 (red). SHX (0.2 mM) was added to the medium at t= 0 min. The vertical dashed lines delimit the different growth phases.

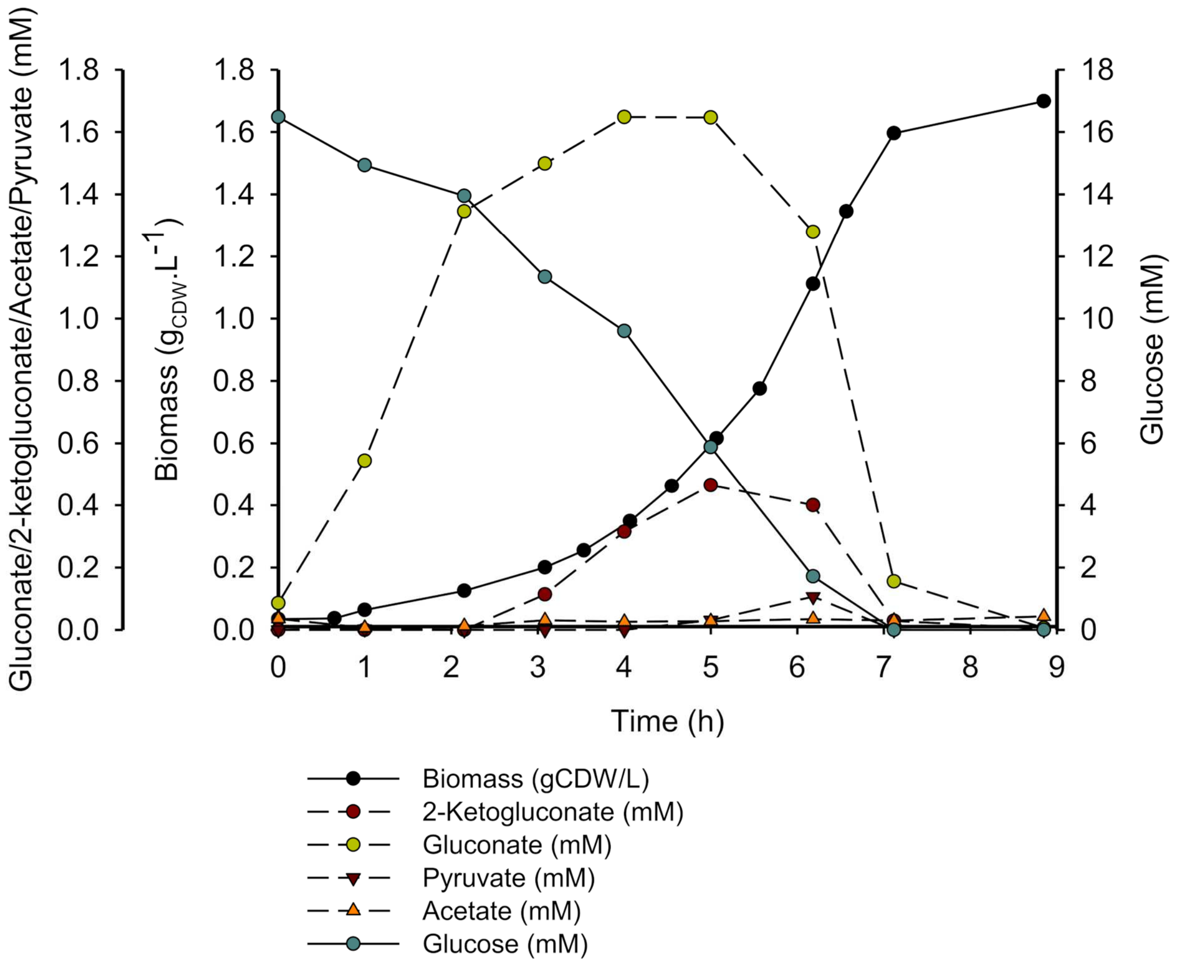

Supplementary Figure S3: Growth profile of wild type *P. putida* KT2440 cultivated in M9 medium supplemented with 3 g·L^{-1} glucose without SHX addition.

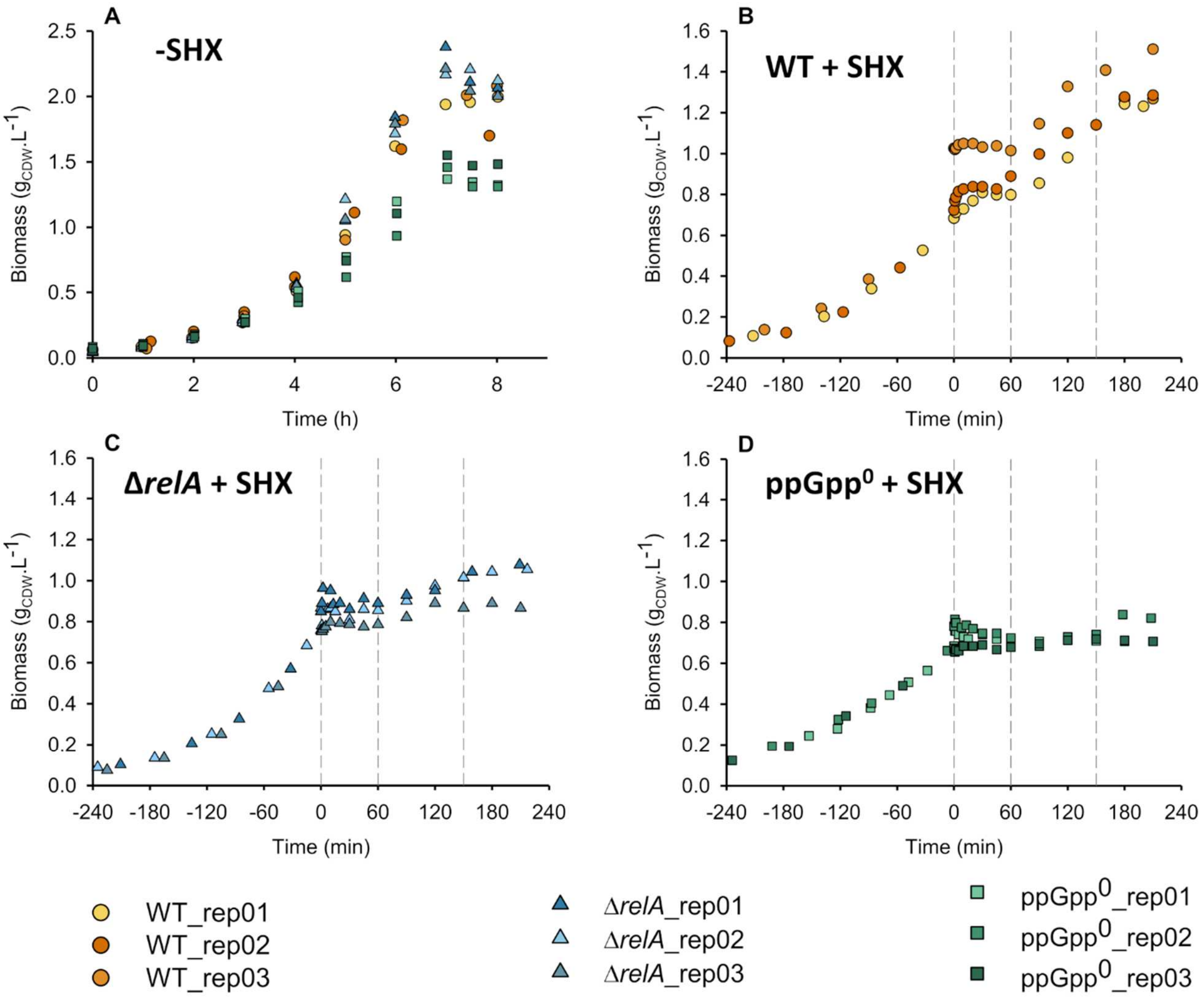

Supplementary Figure S4: Growth curves of the *P. putida* KT2440 WT strain (yellow to dark orange), the Δ*relA* mutant (blue to dark blue) and the ppGpp^0 strain (green to dark green) without SHX (A) and in response to SHX addition (B, C and D) in three independent biological replicates. SHX (0.2 mM) was added to the medium at t= 0 min. The vertical dashes delimit the different growth phases defined for the WT strain.

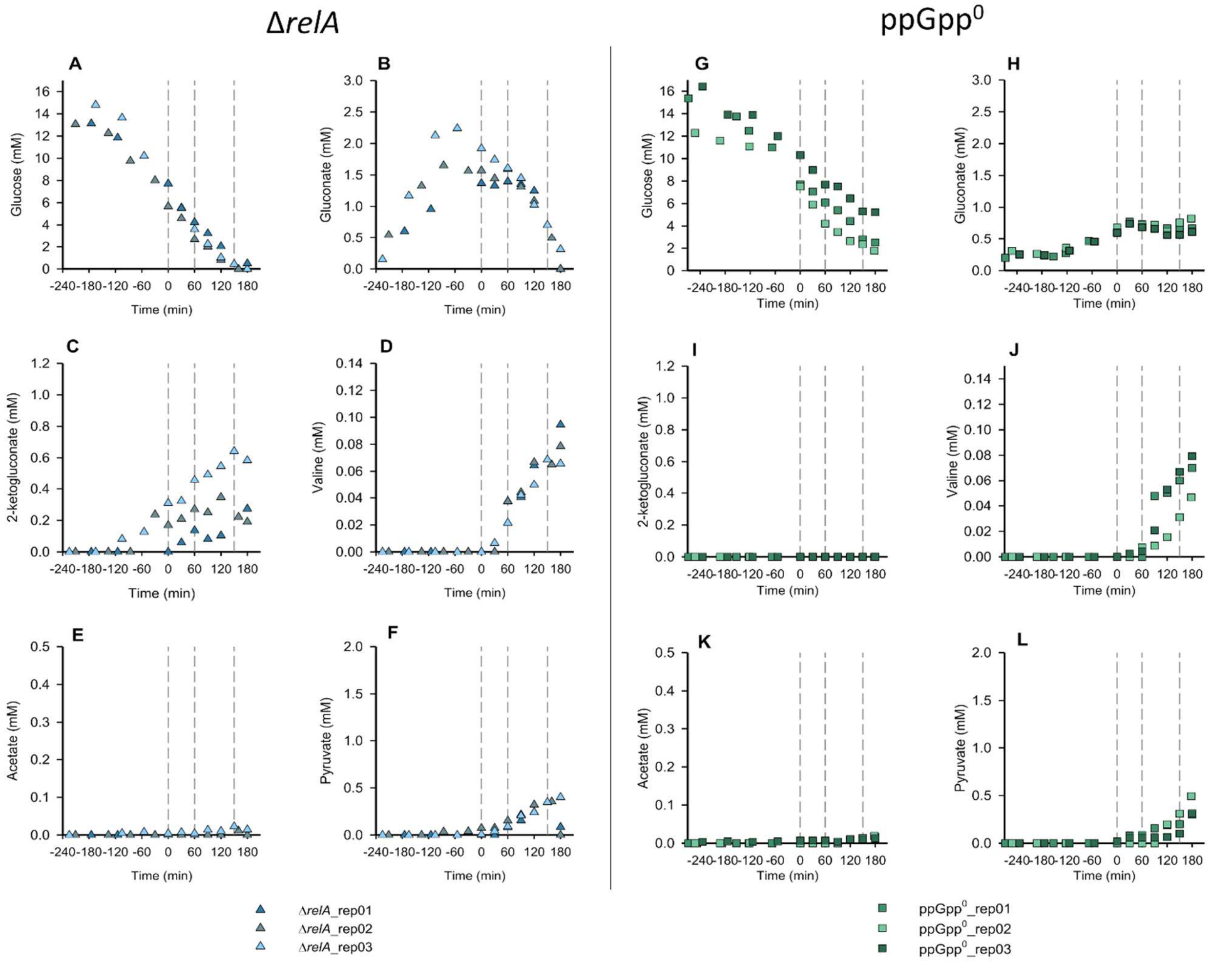

Supplementary Figure S5: Profiles of the extracellular metabolites quantified throughout the cultivations of the *P. putida* KT2440 Δ*relA* mutant (left panel) and ppGpp^0 strain (right panel) in the three independent biological replicates. Time-evolution of glucose (**A,G**), gluconate (**B,H**), 2-ketogluconate (**C,I**), valine (**D,J**), acetate (**E,K**) and pyruvate (**F,L**). SHX (0.2 mM) was added to the medium at t= 0 min. The vertical dashed lines delimit the different growth phases.

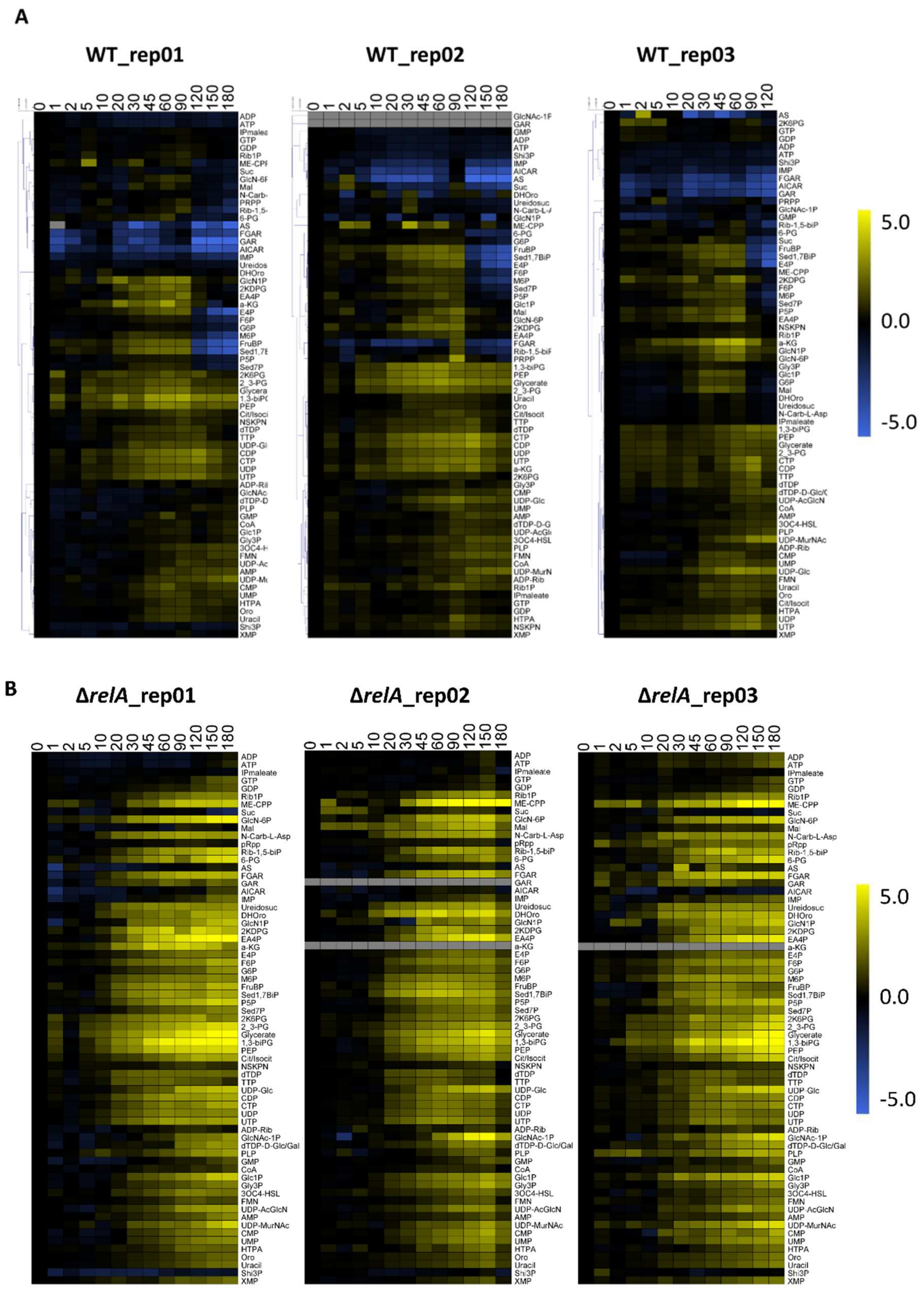
A
WT_rep01
WT_rep02
WT_rep03
5.0
0.0
-5.0
B
ΔrelA_rep01
ΔrelA_rep02
ΔrelA_rep03
5.0
0.0
-5.0

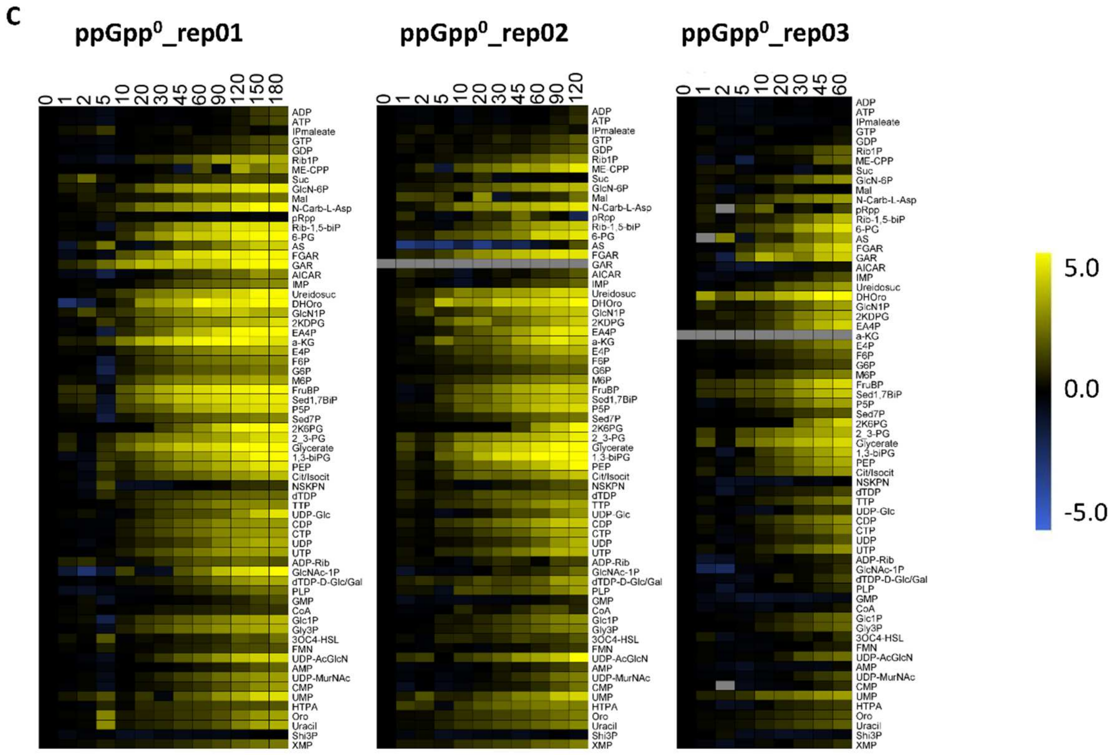

Supplementary Figure S6: Metabolome dynamics in response to SHX addition in wild type (WT) *P. putida* KT2440 (A), the Δ*relA* mutant (B) and the ppGpp^0 strain (C). The data from Figure 4 correspond to rep01. Columns represent different time points just before (t=0 min) and after SHX addition. Metabolome dynamics were established from LC-MS $^{12}C/^{13}C$ quantitative data of 67 metabolites. Fold-change (log_2) is relative to exponentially growing cells. Metabolites from WT strain were hierarchically clustered by using Pearson correlation. For comparison simplicity, metabolites are displayed in same order for the Δ*relA* mutant (**B**) and the ppGpp^0 strain (**C**) as for the WT_rep01 (**D**). Log_2 ratios of the relative metabolite concentration changes for the three biological replicate are provided in Supplementary material. Not detected metabolites by LC-MS are grey. Non-canonical abbreviations: 1,3-biPG: 1,3-biphosphoglycerate; 2_3-PG: 2/3-phosphoglycerate; 2K6PG: 2-keto-6-phosphogluconate; 2KDPG: 2-keto-deoxy-phosphogluconate; 3OC4-HSL: 3-Oxo-N-(2-oxotetrahydro-3-furanyl)butanamide; 6-PG 6-Phosphogluconate; ADP-Rib: ADP-Ribose; AICAR: 5'-Phosphoribosyl-5-amino-4-imidazolecarboxamide; a-KG: α-ketoglutarate; AS: Adenylosuccinate; Cit/Isocit: Citrate/Isocitrate; CoA: Coenzyme A; DHOro: Dihydroorotate; dTDP: deoxy-TDP; dTDP-D-Glc: deoxy-TDP-D-glucose; E4P: Erythrose-4-phosphate; EA4P: 4-Phospho-D-erythronate; F6P: Fructose-6-phosphate; FGAR: 5'-Phosphoribosyl-N-formylglycinamide; FMN: Flavin Mononucleotide; FruBP: Fructose-1,6-bisphosphate ; G6P: Glucose-6-phosphate; GAR: 5'-phosphoribosylglycinamide; Glc1P: Glucose-1-phosphate; GlcN1P: glucosamine-1-phosphate; GlcN-6P: Glucosamine-6-phosphate; GlcNAc-1P: N-acetyl-D-glucosamine 1-phosphate; Gly3P: Glycerol-3-phosphate; HTPA: Tetrahydrodipicolinate; IPmaleate: Isopropylmaleate; M6P: Mannose-6-phosphate; Mal: Malate; ME-CPP: 2-C-Methyl-D-erythritol 2/4-cyclodiphosphate; N-Carb-L-Asp: N-carbamoyl-L-aspartate; NSKPN: N-Succinyl-2-amino-6-ketopimelate; Oro: Orotate; P5P: Pentose-5-phosphate; PEP: Phophoenolpyruvate; PLP: Pyridoxal 5'-phosphate; pRpp: Phophoribosyl pyrophosphate; Rib-1,5-biP: Ribose-1,5-biphosphate; Rib1P: Ribose-1-phosphate; Sed1,7BiP: Sedoheptulose-1,7-bisphosphate; Sed7P: Sedoheptulose-7-phosphate; Shi3P: Shikimate 3 phosphate;

Suc: Succinate; TTP: Thymidine 5'-triphosphate; UDP-AcGlcN: UDP-acetylglucosamine; UDP-Glc: UDP-glucose; UDP-MurNAc: UDP-N-Acetylmuraminate; Ureidosuc: Ureidosuccinate.

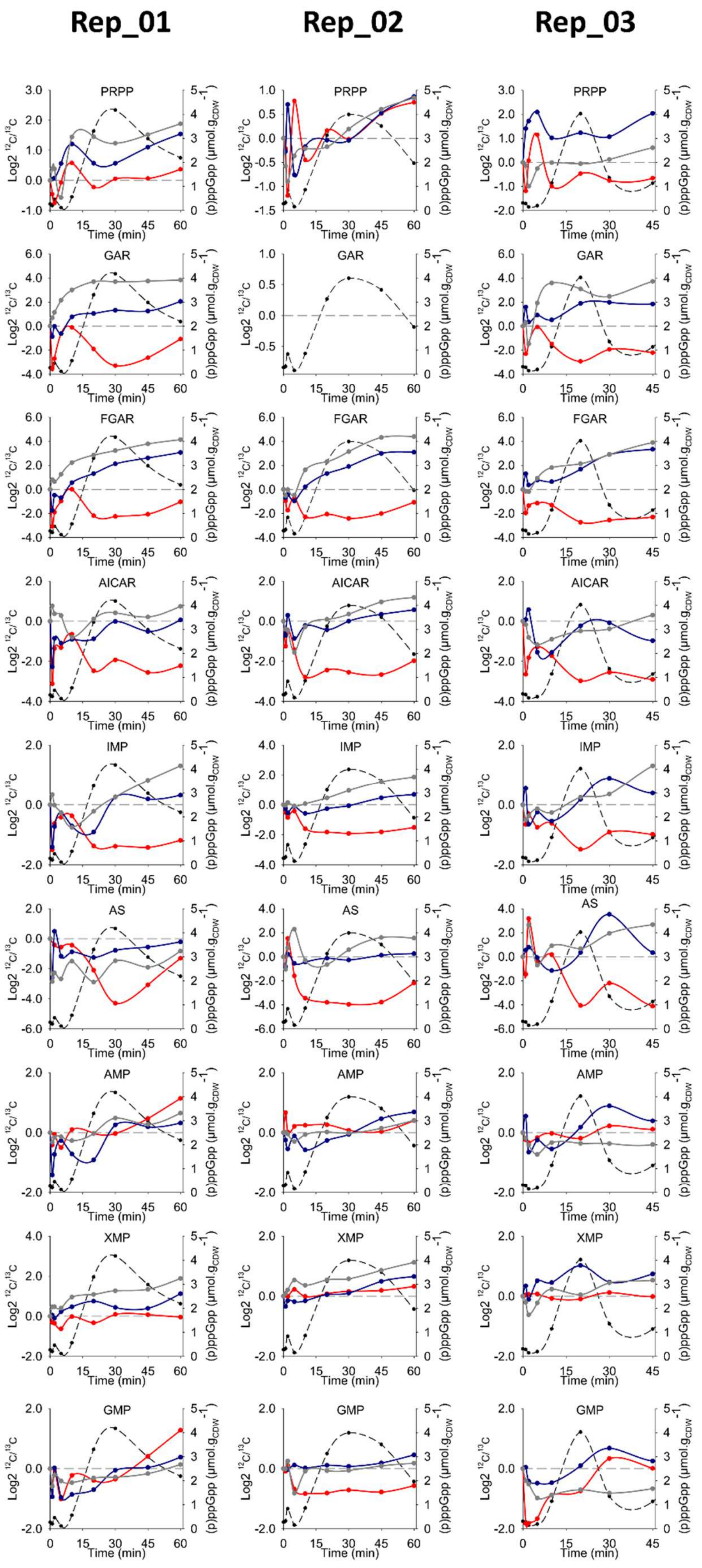
Rep_01
Rep_02
Rep_03
PRPP
GAR
FGAR
AICAR
IMP
AS
AMP
XMP
GMP
Log2 ^{12}C/^{13}C
(p)ppGpp (µmol.g$_{CDW}$$^{-1}$)
Time (min)

Supplementary Figure S7: **Relative quantification of metabolites within the *de novo* purine biosynthesis pathway in the wild type *P. putida* strain (red), the Δ*relA* mutant (blue) and the ppGpp^0 strain (grey), for the three independent biological replicates.** The data from Figure 5 correspond to rep01. Short-term concentration variations are shown during the first phase of (p)ppGpp accumulation (black). Metabolite-concentration fold changes are expressed as $\log_2$ ratios. The dashed grey lines on each graph show the baseline levels (i.e. intracellular levels before SHX addition). Of note, GAR was not detected for rep02. Non-canonical abbreviations: pRpp: phosphoribosylamine; GAR: 5'-phosphoribosylglycinamide; FGAR: 5'-Phosphoribosyl-N-formylglycinamide; AICAR: 5'-Phosphoribosyl-5-amino-4-imidazolecarboxamide; AS: Adenylosuccinate.

Supplementary Table

Supplementary Table S1 Bacterial strains used in this work

Strain	Genotype	Reference
P. putida KT2440	mt-2 *hsdR*1 (r^- m^+)	Díaz-Salazar et al. 2017
Δ*relA*	KT2440-Tel Δ*relA*::Km. Tel^r Km^r	Díaz-Salazar et al. 2017
$ppGpp^0$	KT2440-Tel Δ*relA*::Km Δ*spoT*::Gm. Tel^r Km^r Gm^r	Díaz-Salazar et al. 2017

References

Diaz-Salazar, C., Calero, P., Espinosa-Portero, R., Jimenez-Fernandez, A., Wirebrand, L., Velasco-Dominguez, M. G., et al. (2017). The stringent response promotes biofilm dispersal in Pseudomonas putida. *Sci Rep* 7, 18055. doi:10.1038/s41598-017-18518-0.
